# Addressing Nursing Resignation: Insights From Qualitative Studies on Nurses Leaving Healthcare Organisations and the Profession

**DOI:** 10.1111/jan.16546

**Published:** 2024-10-18

**Authors:** Lara Lessi, Ilaria de Barbieri, Matteo Danielis

**Affiliations:** ^1^ Surgical Department Padua University Hospital Padova Italy; ^2^ Laboratory of Studies and Evidence Based Nursing, Department of Cardiac, Thoracic, Vascular Sciences and Public Health University of Padua Padova Italy; ^3^ Healthcare Professionals Department Padua University Hospital Padua Italy

**Keywords:** meta‐summary, nurse shortage, nurses, qualitative synthesis, resignation, systematic review, turnover

## Abstract

**Aim:**

The aim of this study is to explore the experiences of nurses who resigned from healthcare organisations or abandoned the profession and explore the reasons behind them.

**Design:**

A systematic review of qualitative studies and meta‐summary.

**Data Sources:**

Cumulative Index to Nursing and Allied Health Literature (CINAHL), Embase (Ovid), MEDLINE (Ovid), Social Science Citation Index (Web of Science), and Scopus.

**Methods:**

The search was conducted up to May 2024. Primary qualitative studies focused on nurses who had resigned or left the profession were included. The meta‐summary was conducted using method: findings were extracted from the reports, edited, grouped, abstracted into key meta‐findings, and finally, their frequency effect sizes were calculated.

**Results:**

A total of 282 findings were extracted from 12 studies, generating 49 statements of findings that were aggregated into nine key meta‐findings. Poor management practices presented a frequency effect size of 100%. Other key meta‐findings included excessive workload, teamwork hurdles, health issues related to work shifts and difficulty in maintaining work‐life balance, a lack of career growth opportunities and promotion chances, disillusionment with nursing, dissatisfaction due to salary, bullying and horizontal violence, and moral distress over ethical dilemmas.

**Conclusion:**

The findings can help support the development of targeted strategies and the implementation of effective policies aimed at reducing nursing turnover.

**Impact and Implications for the Profession:**

The major impact of these findings is the recognition of rising factors that negatively affect nurses' quality of life, including workload pressures and poor management strategies, which significantly lower job satisfaction. To address these challenges, the profession should prioritise tools that value nurses in their roles, implement strategies to manage workloads more effectively and advocate for policies promoting flexible scheduling. Additionally, investing in professional development and fostering a supportive work environment can help retain skilled nurses and nurture the growth of new talent.

**Reporting Method:**

Preferred Reporting Items for Systematic Reviews and Meta‐Analyses (PRISMA).

**Patient or Public Contribution:**

No patient or public contribution.

**Summary**

- What does this paper contribute to the wider global clinical community?
  ○ Highlights critical factors such as poor management practices, excessive workloads, and lack of career growth opportunities that contribute to nurse resignations, offering a deeper understanding of the challenges faced by nursing professionals
  ○ Supports the development and implementation of targeted strategies and effective policies aimed at retaining nurses within healthcare organisations and the profession.
  ○ Underscores the multifactorial nature of the nursing shortage, emphasising the need for diversified interventions and informed policy‐making to address this prevalent issue on a global scale.

## 1 Introduction

Nurses are the largest category of healthcare professionals and play a fundamental role in providing care in hospital settings, long‐term care facilities, and the community (WHO 2020). Globally, the shortage of the nursing workforce is intensified by current demographic and health trends. These include an aging population, an increase in chronic diseases and a consequently growing need for healthcare assistance (Buchan, Catton, and Shaffer 2022; OECD and European Union 2022). Additional factors contributing to the shortage of nurses include the insufficient number of students who both begin and complete their nursing education (CREA 2022), the aging of the nursing workforce (Buchan, Catton, and Shaffer 2022; OECD and European Union 2022) and the physical and mental stress associated with the profession, as highlighted in an Italian cross‐sectional survey of 3667 medical and surgical nurses (Sasso et al. 2019). Moreover, there is also a significant number of nurses leaving the profession. Among other factors, the Coronavirus Disease 2019 (COVID‐19) pandemic increased nurses' workload and stress, negatively affecting their intention to leave (Ulupınar and Erden 2024).

The intentions of nurses to quit their profession have been the subject of considerable research attention, both before the spread (Sasso et al. 2019) and during the COVID‐19 pandemic (Raso, Fitzpatrick, and Masick 2021). In fact, 35.5% of nurses intended to leave their current hospital within the next year due to job dissatisfaction, and of these, 33.1% planned to leave the nursing profession altogether (Sasso et al. 2019). These data are confirmed during the COVID‐19 pandemic, where the estimated overall intention to leave the nursing job was 31.7%, ranging from 20.6% to 48.6% (Ulupınar and Erden 2024). It is important to emphasise that nurses' intention to leave their jobs does not necessarily equate to actual turnover; therefore, it is essential to carefully examine the motivations behind the decision to resign to fully understand the decision‐making process.

The data on the nursing shortage and its contributing factors assume critical importance when considering that adequate staffing levels are needed to meet the growing demands for healthcare services, given the fundamental role nurses play in providing direct patient care, promoting health and enhancing patient compliance and satisfaction with healthcare services received (Aiken et al. 2014; Dall'Ora et al. 2022). Deficiencies in both quantitative and qualitative aspects of nursing staffing levels have been linked to adverse patient outcomes, including adverse events in hospital settings, falls in clinical wards, errors in medication administration, a lack of appropriate care practices, delays in hospital discharges (Wang et al. 2020); a significant reduction in perceived patient satisfaction concerning the care received (Winter, Schreyögg, and Thiel 2020) and a rise in mortality rates (Aiken et al. 2014; Dall'Ora et al. 2022). Furthermore, inadequate staffing levels have negative effects on nurses, as they are consistently associated with a higher degree of burnout, increased job dissatisfaction and a higher intent to leave (Shin, Park, and Bae 2018).

Contributing to the overall shortage of the nursing workforce, many nurses have expressed a willingness to leave the profession (Flinkman, Isopahkala‐Bouret, and Salanterä 2013). Researchers have distinguished between external turnover, which involved individuals leaving an organisation for various reasons, and internal turnover, which includes job changes within the organisation (Hayes et al. 2012), even when these involve opportunities for advancement. Nurse turnover is often described as a sequential process. Initially, nurses might leave their specific unit, then move on to leave the organisation, and eventually, they might exit the nursing profession entirely (Flinkman, Leino‐Kilpi, and Salanterä 2010). Mild forms of turnover, such as leaving a ward or organisation, often precede more severe outcomes like leaving the nursing profession altogether (Tummers, Groeneveld, and Lankhaar 2013). When employees indicate their intention to leave, it typically suggests that they are considering an external turnover, meaning they are thinking about leaving the organisation for a new position or career change.

In the literature, the intention to leave is a key predictor of nurse turnover and represents a cognitive process involving thoughts, planning and decision‐making prior to actually leaving (Tadesse et al. 2023). This issue is prevalent globally, affecting nurses across various healthcare settings, including hospitals, community home care and nursing homes (Tummers, Groeneveld, and Lankhaar 2013). Over the past 20 years, research has extensively examined this concept. Coomber and Barriball (2007) identified four job characteristics most related to the intent to leave and turnover among hospital‐based nurses: leadership, development opportunities, stress and salary. Similar findings regarding management style, development opportunities and work pressure were reported in a systematic review by Hayes et al. (Hayes et al. 2006), with an update in 2012 emphasising factors such as management style, workload and empowerment (Hayes et al. 2012). The combination of those intending to leave and those uncertain about their future positions could destabilise the workforce if not addressed (Raso, Fitzpatrick, and Masick 2021). Additionally, the workload of nurses, which contributes to missed care, is linked to an increased intention to leave, mediated by job satisfaction (Alsubhi et al. 2020). Cultural and social factors, such as the undervaluing of nursing care and discrimination, also play a significant role (Mohammadi et al. 2023).

Studies have shown that the intention to leave is a predictor of the final decision to leave the profession (Hayes et al. 2006). Through the years, the concept of intention to leave has been extensively examined both with quantitative and, to a lesser extent, qualitative research designs (Flinkman, Leino‐Kilpi, and Salanterä 2010). Although most turnover research has concentrated on nurses' intentions to quit their current job or organisation, there is less literature on the factors that drive nurses to leave the profession altogether (Flinkman, Leino‐Kilpi, and Salanterä 2010). Bahlman‐van Ooijen et al. (2023), in a recent meta‐aggregation of qualitative studies, identified four main themes regarding the motivations behind the intention to leave the nursing profession: a challenging work environment, emotional distress, disappointment about nursing reality and a culture of hierarchy and discrimination.

Despite the abundance of studies on intention to leave, limited attention has been paid to studying the final stages of the turnover process, where nurses resign from their organisation or leave the nursing profession. Due to the subjective and psychological nature of the turnover phenomenon, it is crucial to gain a comprehensive understanding of these final stages. Qualitative research in this context provides a unique opportunity to thoroughly explore the human experience and fully understand the complex dynamics that contribute to nursing turnover.

Using a qualitative meta‐summary methodology, this study sought to provide a comprehensive overview of the available international research to gain an understanding of the motivations that drive nurses to leave their organisations and the profession, thus offering an integrated description of the phenomenon. Fully understanding this phenomenon is the first step in developing targeted strategies that can help healthcare organisations fight nurse turnover and promote a more sustainable and rewarding work environment for nurses.

## 2 The Review

### 2.1 Aim

This review aims to provide an overview of the evidence from qualitative studies that delve into the experiences of nurses who have left healthcare organisations and/or abandoned the profession and explore the reasons behind this final decision.

Research Question: What are the motivations that lead nurses to resign from healthcare organisations or leave the nursing profession, as explored in qualitative studies?

### 2.2 Design

This qualitative research synthesis (i.e., the systematic and integrated interpretation of findings from previous qualitative studies) was conducted following the method proposed by Sandelowski and Barroso (2007). The study follows four stages: (1) formulating the research question and aim, (2) conducting a literature search, (3) appraising reports and (4) synthesising qualitative research findings (meta‐summary) (Sandelowski and Barroso 2007).

The findings of this synthesis are reported here following the Enhancing Transparency in Reporting the Synthesis of Qualitative Research (ENTREQ) statement (Tong et al. 2012). Moreover, this synthesis adhered to a predetermined protocol that was prospectively registered in the International Prospective Register of Systematic Reviews database (PROSPERO) under registration number CRD42023491582.

### 2.3 Search Methods

The research was conducted using the following databases: Cumulative Index to Nursing and Allied Health Literature (CINAHL), Embase (Ovid), MEDLINE (Ovid), Social Science Citation Index (Web of Science) and Scopus, for literature published up to May 2024 (last search on May 15, 2024).

The search strategy was developed using the SPIDER Tool (Cooke, Smith, and Booth 2012): S (Sample) nurses, PI (Phenomenon of Interest) resignation, D (Design) not specified, E (Evaluation) motivations and R (Research type) qualitative research. The keywords ‘nurse’, ‘motivation’, ‘reason’, ‘resignation’, ‘quitting’, ‘leave’, ‘turnover’ and ‘qualitative research’ were combined into specific search strings (Table 1). This tool provides a systematic approach for locating qualitative research studies. Similar to the PICO (Patient/Population, Intervention, Comparison, Outcomes) tools, the SPIDER tool enhances research rigour by outlining essential components of non‐quantitative research questions (Methley et al. 2014).

**TABLE 1:** Search strategy.

Database	Search query	Results	Date of last search
CINAHL	nurs* AND (motivation or reason) AND (resignation or quit* or leav* or turnover) AND qualitative	480	15/05/24
SCOPUS	(TITLE‐ABS‐KEY (nurs*) AND TITLE‐ABS‐KEY (motivation OR reason) AND TITLE‐ABS‐KEY (resignation OR quit* OR leav* OR turnover) AND TITLE‐ABS‐KEY (qualitative))	314	15/05/24
Web of science	nurs* (All Fields) and motivation OR reason (All Fields) and resignation OR quit* OR leav* OR turnover (All Fields) and qualitative (All Fields)	340	15/05/24
Embase	nurs* (Abstract) AND motivation OR reason (All Fields) AND resignation OR quit* OR leav* OR turnover (Abstract) AND qualitative (All Fields)	223	15/05/24
Medline	nurs* (Abstract) AND motivation OR reason (All Fields) AND resignation OR quit* OR leav* OR turnover (Abstract) AND qualitative (All Fields)	100	15/05/24

### 2.4 Inclusion and Exclusion Criteria

This qualitative synthesis encompasses primary studies that utilise qualitative methods such as phenomenology, grounded theory, ethnography and descriptive research. Surveys that incorporate qualitative components, like open‐ended or essay questions, were also considered for inclusion. The synthesis included studies that focused on nurses who had resigned from healthcare organisations or had left the nursing profession, regardless of their age, level of experience or specific affiliations. Moreover, studies that solely examined the intention to leave, involving nurses who were still employed at the time of the study, were excluded. Studies that directly reported on the experiences of this population were included, whereas those that approached the phenomenon from other perspectives were excluded. Only studies published in English were included. A timeframe was not chosen because the authors wanted to explore the phenomenon in a comprehensive manner, beyond the constraints of time.

### 2.5 Search Outcome

**FIGURE 1:**
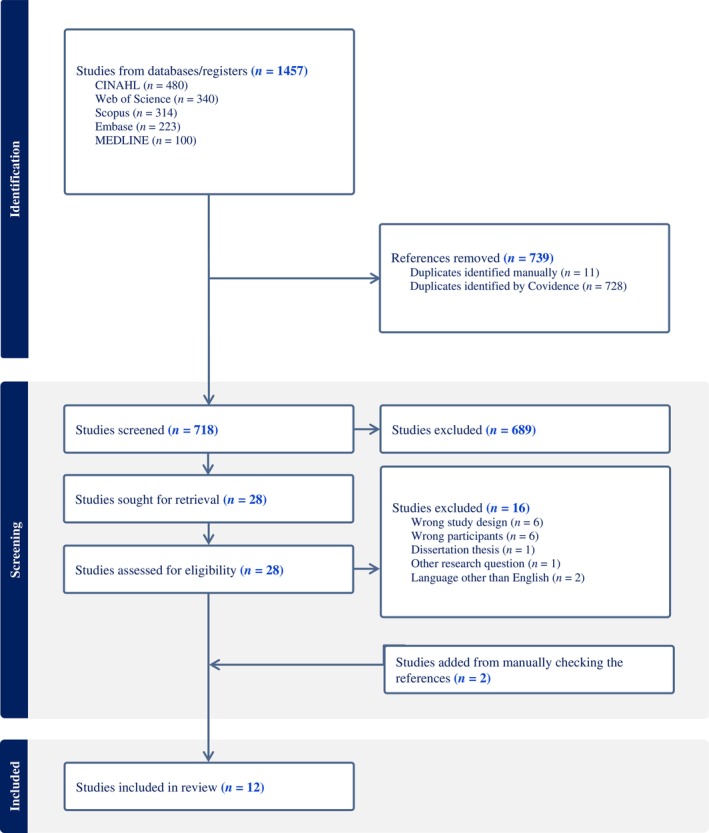
Flow chart of the search strategy and results.

The search identified a total of 1457 articles. All the articles were imported into the Covidence platform, which helped with document management and the removal of duplicates. Once duplicates were removed, the titles and abstracts of 718 articles were independently screened by two researchers, leaving 29 relevant articles, which were fully read and evaluated. The selection of articles was based on predetermined inclusion and exclusion criteria, and any disagreements were discussed until consensus was reached. Finally, 10 articles were included in the synthesis. Furthermore, the reference lists of all eligible studies were examined to identify other potentially eligible primary studies. Those that could be obtained in full text were then screened for eligibility using the aforementioned criteria. Two articles were added after manually checking the references of the selected studies. An overview of the screening and selection process is shown in Figure 1.

### 2.6 Quality Appraisal

The methodological quality of the included studies was assessed with the Critical Appraisal Skills Programme (CASP) qualitative study checklist (CASP 2023). Each of the 10 items was rated from 0 to 1 (0 criterion not met, 0.5 criterion partially met and 1 criterion met). According to previous studies (Danielis et al. 2024), studies were considered to achieve lower (CASP 0–5.5), medium (CASP 6–8.5) or higher (CASP 9–10) levels of quality. No report was excluded for reasons of methodological quality. This is in line with Sandelowski & Barroso (Sandelowski and Barroso 2003), who argue that no consensus exists concerning quality, neither in qualitative research nor in the use of quality criteria in systematic reviews (Herber et al. 2017). Each article was initially critically appraised independently by two authors. Any disagreements were then discussed among all three authors to reach a final consensus.

### 2.7 Data Extraction

The data were transferred to a Microsoft Excel spreadsheet. The following data were extracted from each study and reported in the grid: author, year, country of origin, aim, sample, data collection method, stated theoretical approach, analysis technique and key themes.

Subsequently, the process of synthesising the findings was initiated following the method proposed by Sandelowski and Barroso (2007). The findings were defined and separated within the articles by: (a) data presentations: quotes, stories, and cases used by researchers to support their results; (b) data and findings not about the target area; (c) findings imported from other studies referenced by the researchers; (d) analytical procedures, such as descriptions of coding methods and data visualisations and (e) researchers' discussions on the significance, implications, and relevance of their findings for research, education, practice or policy making (Sandelowski and Barroso 2007). This process was carried out using the ATLAS.TI software, where all the included studies were imported.

Once the findings were extracted, they were edited to make them as accessible as possible to any reader, keeping as close as possible to the authors' words and preserving their intentions as required by the Sandelowski and Barroso (2007) method. Then, three researchers independently grouped findings that appeared to be about the same topic and formulated the statements of findings. They discussed to reach consensus on each statement.

In the abstraction process, statements of findings were combined into more concise renderings, following the same consensus process involving the three researchers. At the end of the abstraction process, the key meta‐findings were identified as a set of statements that concisely but comprehensively captured the content of all the findings and preserved the context in which they appeared (Sandelowski and Barroso 2007).

An inter‐study matrix was used to calculate the effect sizes of frequency and intensity of the abstracted findings. The frequency effect size was calculated by dividing the number of reports containing the finding by the total number of reports. The intensity effect size was calculated by comparing the number of findings in each study with the total number of findings in all reports (Sandelowski and Barroso 2007). In Results section, the findings are presented in order of frequency effect size.

## 3 Results

### 3.1 Characteristics of the Studies

The years of the publications of the 12 selected studies range from 2006 (King and McInerney 2006) to 2024 (Bäckström, Pöder, and Karlsson 2024; Pyhäjärvi and Söderberg 2024); the countries included Australia (Jarden et al. 2023), China (Hung and Lam 2020; Zhu et al. 2021; Zhu, Rodgers, and Melia 2014), Turkey (Çamveren, Arslan Yürümezoğlu, and Kocaman 2020), South Africa (King and McInerney 2006), Sweden (Lögde et al. 2018; Bäckström, Pöder, and Karlsson 2024), the Netherlands (Kox et al. 2020), Canada (Chachula, Myrick, and Yonge 2015), the United States (MacKusick and Minick 2010) and Finland (Pyhäjärvi and Söderberg 2024). Sample sizes ranged from 8 (Chachula, Myrick, and Yonge 2015) to 39 (Jarden et al. 2023) resigned nurses, with a median sample size of 16. The demographic characteristics of the participants included in these studies mainly comprised female nurses, as shown in Table 2. One study (Bäckström, Pöder, and Karlsson 2024) did not provide a gender breakdown of the sample.

**TABLE 2:** Characteristics of the included studies.

Authors/Year/Country	Aim	Setting	Sample/Sex/Age	Data collection	Stated theoretical approach/data analysis	Key themes/Subthemes
Jarden et al. (2023) Australia	To explore and describe the work well‐being experiences and perceptions of nurses who resigned, starting from what inspired them to join their organisations, what created a positive workday for them, to what might have supported them in staying	Acute and sub‐acute care, clinics, and community settings	39 nurses (33 ♀, 6 ♂)	Semi‐structured interviews	Not Reported	COVID‐19
					Thematic analysis by Braun & Clarke (2022)	Uncertainty of role
			Median age 42 years			Workload and rostering (also known as shift scheduling)
						Not feeling supported, respected, and valued
Hung and Lam (2020) China	To identify the antecedents and contextual factors that have contributed to the decisions of occupational turnover from their clinical duties among registered nurses in public hospitals in Hong Kong	Public hospitals in Hong Kong	18 nurses (14 ♀, 4 ♂)	Semi‐structured interviews	Not Reported	Job dissatisfaction due to a tense work environment:
						Excessive and additional workload, strained relationships with colleague
			Age 21‐ over 50 years		Content analysis by Graneheim & Lundman (2004)	Low motivation due to limited career opportunities: scanty professional development, poor career advancement opportunities
						Inadequate communication due to ineffective leadership: inattentive administration and management, unfair treatment
Çamveren, Arslan Yürümezoğlu, and Kocaman (2020) Turkey	To reveal the reasons for young nurses' leaving their organisation by reflecting on their own experiences and perspectives	Public university hospital in Turkey	15 nurses (14 ♀, 1 ♂)	Semi‐structured interviews	Not Reported	Negative work environment
			Age 24–29 years		Content analysis method and the three‐step analysis process developed by Miles & Huberman (1994)	
						Nursing shortage
						Unsatisfied individual expectations
Zhu et al. (2021) China	To explore the experiences of resigned nurses with career plateau	Hospital in Beijing	9 nurses (8 ♀, 1 ♂)	Semi‐structured interviews	Husserl phenomenological research	Extremely disappointed with current work
					Colaizzi method to highlight the text code, The Qualitative Analysis Guide and steps of phenomenological analysis by LoBiondo‐Wood (2002)	
			Age 29–38 years			
						Deeply uncertain about the future
						Unsatisfied personal needs
						Lack of organisational support
King & Mcinerney (2006) South Africa	To explore and describe the hospital workplace experiences that had contributed to the resignations of Registered Nurses in the Durban Metropolitan Area	Four hospitals in the Durban Metropolitan Area	15 nurses (13 ♀, 2 ♂)	Semi‐structured interviews	Phenomenological approach	Working conditions and experience
			Age not reported		Phenomenological analytical procedures (meaning condensation, coding of natural meaning units and categorization)	Salary, remuneration and other structures
						Interpersonal relationships in the workplace
						Nursing and the nursing profession
						Resignation—recourse to enact personal and positive change
Lögde et al. (2018) Sweden	To identify reasons why specialist nurses in perioperative care chose to leave their workplace and to describe the process from the thought to the decision	Three university hospitals and four county hospitals in Sweden	20 nurses (12 ♀, 8 ♂)	Semi‐structured interviews	Phenomenological approach	The head nurses' betrayal and dismissive attitude and not feeling needed
			Age 28–54 years		Systematic text condensation by Malterud (2012) influenced by Thematic analysis by Braun & Clarke (2012)	Inhumane working conditions leading to the negative health effects
						Not being free to decide about one's life and family life being more important than work
						Colleagues' diminishing behaviour
Kox et al. (2020) Netherlands	To unravel Dutch former novice nurses' reasons, experiences and the circumstances that contributed to their professional turnover within 2 years after graduation	Urban and rural areas in The Netherlands	17 nurses (16 ♀, 1 ♂)	Semi‐structured interviews	Not reported	Lack of challenge
			Age 21–40 years		Thematic analysis by Braun & Clarke (2012)	Lack of passion
						Lack of perceived competence
						Lack of job satisfaction due to heavy workload
						Lack of work capacity due to non‐work‐related health conditions
						Lack of feeling of belonging
Chachula, Myrick, and Yonge (2015)	To explore the factors and basic psychological process involved in the decision of newly graduated registered nurses who permanently exit the nursing profession in western Canada	Western Canada	8 nurses (7 ♀, 1 ♂)	Semi‐structured interviews	Grounded Theory	Overcoming constraints of the healthcare system and workplace
			Age 26–34 years		Grounded theory approach by Glaser (1978)	Negotiating social relationships, hierarchies, and problematic behaviours
						Facing fears, traumas, and challenges
						Balancing competing rewards and tensions
Zhu, Rodgers, and Melia (2014) China	To understand why nurses leave nursing practice by exploring the decision‐making process of registered nurses who have exited clinical care in China	One provincial capital city in the east of China	17 nurses (16 ♀, 1 ♂)	Semi‐structured interviews	Grounded Theory	Entering nursing with unrealistic expectations: choosing nursing with collective expectations, restricting realistic expectations of nursing in education
						Working in the ideal workplace: entering the ideal workplace, committing to the organisation, struggling with a professional identity
						Losing confidence in the safety and quality of health care: perceiving the risk in clinical practice, recognising the organisational barriers to safety, failing to meet expectations of patients
						Nursing autonomy vs. medical dominance: comparing rewards with doctors, struggling with medical dominance
					Comparative analysis by Glaser & Strauss (1967)	Professional value vs. managerial value: emphasising nurses as replaceable labour, losing enthusiasm in promotion, struggling to meet career progress
			Age not reported			Personal freedom vs. organisational control: lack of reasonable nursing mobility, limited maternity leave and sick leave
MacKusick and Minick (2010) United States	To identify the factors influencing the decision of RNs to leave clinical nursing practice	Not specified	10 nurses (8 ♀, 2 ♂)	Semi‐structured interviews	Phenomenological approach	Unfriendly workplace
			Age 22–59 years		Interpretive analysis by Geanellos (2000)	Emotional distress related to patient care
						Fatigue and exhaustion
Bäckström, Pöder, and Karlsson (2024) Sweden	To explore why RNs in Sweden choose to quit their jobs in hospitals, also in relation to patient safety	A university hospital and one county hospital in two healthcare regions in Sweden	11 nurses (Sex not reported)	Semi‐structured interviews	Not reported	Feeling that the profession is not valued
			Age 26–63 years		Systematic text condensation by Malterud (2012)	Psychological and physical symptoms related to work
						An insufficient and unsupportive organisation
						Unsatisfying leadership and teamwork
Pyhäjärvi and Söderberg (2024) Finland	To deepen our understanding of why nurses, decide to leave their occupation instead of changing jobs, we examined the antecedents that led to this decision through the theoretical lens of psychological contract breach	Different types of healthcare organisations, both in the public and private sectors, and lived in different regions of Finland	28 nurses (25 ♀, 3 ♂)	Semi‐structured interviews	Not reported	Little or no support from colleagues
			Age 27–55 years		Thematic analysis by Braun & Clarke (2006)	Ethical dilemmas and moral conflicts related to professionality
						Disappointment in salary
						Not getting support from managers

Three of the studies involved novice nurses (Çamveren, Arslan Yürümezoğlu, and Kocaman 2020; Kox et al. 2020); one study focused on specialist nurses resigned from perioperative care (Lögde et al. 2018); the remaining studies involved resigned nurses from various contexts, such as university hospitals and rural hospitals (Bäckström, Pöder, and Karlsson 2024; Çamveren, Arslan Yürümezoğlu, and Kocaman 2020).

In terms of data analysis methods, the studies used various qualitative approaches to investigate nursing turnover. Jarden et al. (2023), King and McInerney (2006) and Kox et al. (2020) used thematic analysis. Hung and Lam (2020) and Çamveren, Arslan Yürümezoğlu, and Kocaman (2020) used content analysis. Zhu et al. (2021) conducted a phenomenological analysis, whereas Lögde et al. (2018) utilised systematic text condensation. Chachula, Myrick, and Yonge (2015) and Zhu, Rodgers, and Melia (2014) adopted grounded theory. Pyhäjärvi and Söderberg (2024) used reflexive thematic analysis, and MacKusick and Minick (2010) used interpretive analysis. The characteristics of the studies are detailed in Table 2.

As stated in the method section, all studies included in the quality appraisal, using the CASP Checklist, were retained for analysis. However, certain limitations were identified during the assessment of specific elements. Hung and Lam (2020) and Zhu, Rodgers, and Melia (2014) scored lower in the recruitment strategies. King and McInerney (2006), Lögde et al. (2018), Chachula, Myrick, and Yonge (2015), Çamveren, Arslan Yürümezoğlu, and Kocaman (2020) and Zhu, Rodgers, and Melia (2014) were lacking in adequately considering the relationship between researchers and participants. MacKusick and Minick (2010) and Zhu, Rodgers, and Melia (2014) scored low in exposing data collection methods. Although all studies generally had clear statements of research aims, findings and value of the research, there were variations in the rigour of data analysis techniques used. The quality appraisal of the included studies is detailed in Table 3.

**TABLE 3:** Quality assessment of included studies using the Critical Appraisal Screening Programme (CASP 2023).

Items/report	Jarden et al. (2023)	Hung and Lam (2020)	Çamveren, Arslan Yürümezoğlu, and Kocaman (2020)	Kox et al. (2020)	MacKusick and Minick (2010)	Lögde et al. (2018)	Zhu et al. (2021)	King and McInerney (2006)	Chachula, Myrick, and Yonge (2015)	Zhu, Rodgers, and Melia (2014)	Bäckström, Pöder, and Karlsson (2024)	Pyhäjärvi and Söderberg (2024)
1. Was there a clear statement of the aims of the research?	1	1	1	1	1	1	1	1	1	1	1	1
2. Is a qualitative methodology appropriate?	1	1	1	1	1	1	1	1	1	1	1	1
3. Was the research design appropriate to address the aims of the research?	1	1	1	1	1	1	1	1	1	1	0,5	1
4. Was the recruitment strategy appropriate to the aims of the research?	0,5	1	1	1	1	1	1	1	1	0,5	1	1
5. Was the data collected in a way that addressed the research issue?	1	1	1	1	1	1	1	0,5	1	0,5	1	1
6. Has the relationship between researcher and participants been adequately considered?	1	0	1	0,5	0	0,5	1	0	0	0	1	1
7. Have ethical issues been taken into consideration?	1	1	1	1	0,5	1	1	1	1	1	1	1
8. Was the data analysis sufficiently rigorous?	1	1	1	1	0,5	1	1	0,5	1	1	1	1
9. Is there a clear statement of findings?	1	0,5	1	1	0,5	1	1	1	0,5	1	1	0,5
10. How valuable is the research?	1	1	1	1	1	0,5	0,5	1	1	1	1	1
Overall score	9,5	8,5	10	9,5	7,5	9	9,5	8	8,5	8	9,5	9,5
Overall level of methodological quality	High	Medium	High	High	Medium	High	High	Medium	Medium	Medium	High	High

*Note*: Each element has been evaluated from 0 to 1 (0 = No, 0,5 = Can't tell, and 1 = Yes).

### 3.2 Meta‐Summary

**FIGURE 2:**
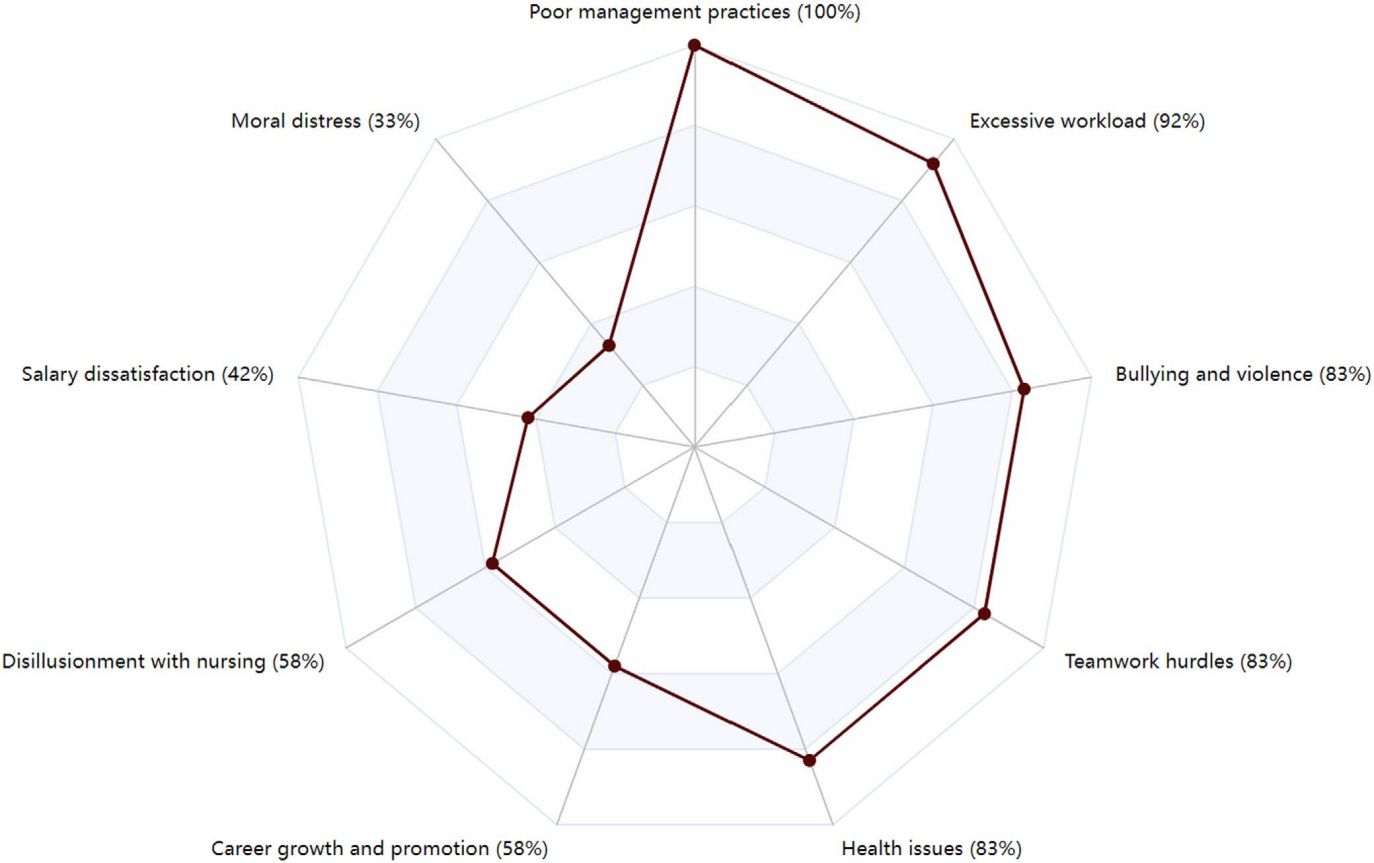
Radar chart of key reasons for nurse resignation and their impact percentages.

As shown in Table 4, all 12 reports contained extractable findings relevant to the research question, which formed the main data for this study. From these studies, a total of 282 findings were extracted, generating 49 statements of findings, which were later grouped into nine key meta‐findings (Figure 2).

**TABLE 4:** Statements of findings organised according to key meta‐findings and frequency effect sizes.

Key meta‐findings and statements of findings combined	Interstudy frequency effect size
Poor management practices, including lack of support, ineffective leadership, and unfair treatment within the healthcare organisation	100%
Nurses felt unsupported by managers in resolving workplace difficulties and supporting professional development (Bäckström, Pöder, and Karlsson 2024; Hung and Lam 2020; Jarden et al. 2023; King and McInerney 2006; MacKusick and Minick 2010; Pyhäjärvi and Söderberg 2024; Zhu, Rodgers, and Melia 2014; Zhu et al. 2021)	
Nurses experienced stress and a lack of perceived competence when they received insufficient support or when they were outsourced to other wards (Bäckström, Pöder, and Karlsson 2024; Çamveren, Arslan Yürümezoğlu, and Kocaman 2020; Kox et al. 2020; MacKusick and Minick 2010; Pyhäjärvi and Söderberg 2024)	
Nurses reported unequal and unfair treatment and decisions regarding the allocation of roles, opportunities, duties, and shifts (Hung and Lam 2020; Jarden et al. 2023; King and McInerney 2006; Lögde et al. 2018)	
Nurses reported an ineffective leadership style, which affected communication, lack of listening to or ignoring employees' concerns, not being taken seriously, making decisions without employee involvement, and not having the employees' interests in mind (Bäckström, Pöder, and Karlsson 2024; Chachula, Myrick, and Yonge 2015; Hung and Lam 2020; Lögde et al. 2018)	
Nurses felt undervalued and unappreciated in their experiences and competencies (Bäckström, Pöder, and Karlsson 2024; Hung and Lam 2020; Jarden et al. 2023; Kox et al. 2020)	
Nurses felt disappointed and frustrated towards managers, coupled with dissatisfaction with staffing practices (King and McInerney 2006; Kox et al. 2020; Pyhäjärvi and Söderberg 2024)	
Nurses felt replaceable and undervalued in the organisation (Lögde et al. 2018; Pyhäjärvi and Söderberg 2024; Zhu, Rodgers, and Melia 2014)	
Nurses reported the healthcare organisation as passive and rigid (Chachula, Myrick, and Yonge 2015; Hung and Lam 2020)	
Nurses reported subjective process utilised in performance appraisals (King and McInerney 2006)	
Excessive workload, worsened by staff shortages, role ambiguity, COVID‐19, additional duties such as training, and unclear role boundaries	92%
Nurses reported an excessive workload due to staff shortages and continuous requests to work overtime (Bäckström, Pöder, and Karlsson 2024; Çamveren, Arslan Yürümezoğlu, and Kocaman 2020; Hung and Lam 2020; Kox et al. 2020; Lögde et al. 2018; MacKusick and Minick 2010; Pyhäjärvi and Söderberg 2024; Zhu, Rodgers, and Melia 2014)	
Nurses reported that excessive workload and stress led to critical incidents and concerns about patient safety (Bäckström, Pöder, and Karlsson 2024; Chachula, Myrick, and Yonge 2015; Kox et al. 2020; Pyhäjärvi and Söderberg 2024)	
Nurses expressed frustration at not being able to provide the quality of care they wish to deliver and feeling like they haven't done enough (Bäckström, Pöder, and Karlsson 2024; Kox et al. 2020; Pyhäjärvi and Söderberg 2024; Zhu, Rodgers, and Melia 2014)	
Intense workloads led nurses to experience significant stress, resulting in both psychological and physical symptoms such as fatigue, sleep disturbances, headaches, muscle tension, lack of confidence, and difficulty relaxing, which negatively impacted their health, well‐being, and ability to recover after work. (Bäckström, Pöder, and Karlsson 2024; Hung and Lam 2020; Kox et al. 2020)	
Nurses reported dissatisfaction attributed to stressful work environment (Chachula, Myrick, and Yonge 2015; Hung and Lam 2020; King and McInerney 2006)	
Nurse reported additional workload stemming from the ambiguous scope of practice and unclear role boundaries (Hung and Lam 2020; Zhu, Rodgers, and Melia 2014)	
Nurse reported poor working conditions (Bäckström, Pöder, and Karlsson 2024; Lögde et al. 2018)	
Nurses reported heavy workload (Hung and Lam 2020; Lögde et al. 2018)	
Nurses reported how COVID‐19 negatively impacted their workload (Jarden et al. 2023; Pyhäjärvi and Söderberg 2024)	
Nurses with junior status found it challenging to handle additional duties, such as training new staff. That negatively impacted their workload (Bäckström, Pöder, and Karlsson 2024; Hung and Lam 2020)	
Bullying, horizontal, and vertical violence, gender‐based abuse, sexual harassment, patient and relative violence, and fear of repercussions for speaking out	83%
Nurses experienced bullism and horizontal violence at work (Çamveren, Arslan Yürümezoğlu, and Kocaman 2020; Chachula, Myrick, and Yonge 2015; King and McInerney 2006; Hung and Lam 2020; Lögde et al. 2018; Kox et al. 2020; MacKusick and Minick 2010; Pyhäjärvi and Söderberg 2024)	
Nurses experienced patient violence, or violence by the patients' relatives (King and McInerney 2006; Kox et al. 2020; Pyhäjärvi and Söderberg 2024)	
Nurses experienced gender‐based abuse and sexual harassment, with perceived acceptance of this behaviour by managers and colleagues (MacKusick and Minick 2010; Pyhäjärvi and Söderberg 2024)	
Nurses experienced vertical violence at work (Çamveren, Arslan Yürümezoğlu, and Kocaman 2020; Zhu, Rodgers, and Melia 2014)	
Nurses felt scared of the consequences of speaking to managers (Jarden et al. 2023)	
Teamwork hurdles, including lack of collaboration, support, understanding, negative relationships, senior resistance to change, and medical dominance	83%
Nurses reported struggling with medical dominance (Bäckström, Pöder, and Karlsson 2024; Chachula, Myrick, and Yonge 2015; King and McInerney 2006; Pyhäjärvi and Söderberg 2024; Zhu, Rodgers, and Melia 2014)	
Nurses reported experiencing negative interpersonal relationships among colleagues, contributing to workplace tension, dissatisfaction, and a challenging work environment (Hung and Lam 2020; King and McInerney 2006; Kox et al. 2020; Lögde et al. 2018; Pyhäjärvi and Söderberg 2024)	
Nurses felt a lack of understanding, acceptance, and support from their colleagues (Bäckström, Pöder, and Karlsson 2024; Jarden et al. 2023; Kox et al. 2020; MacKusick and Minick 2010)	
Nurses expressed lack of team collaboration (Bäckström, Pöder, and Karlsson 2024; Zhu, Rodgers, and Melia 2014)	
Nurses reported reluctance to change by senior colleagues (Kox et al. 2020)	
Health issues related to work shift and difficulty in maintaining work‐life balance	83%
Nurses reported the negative effects that work had on their private lives and the difficulty in maintaining work‐life balance (Bäckström, Pöder, and Karlsson 2024; Çamveren, Arslan Yürümezoğlu, and Kocaman 2020; King and McInerney 2006; Lögde et al. 2018; Kox et al. 2020; Pyhäjärvi and Söderberg 2024; Zhu, Rodgers, and Melia 2014)	
Nurses reported emotional and physical exhaustion (Bäckström, Pöder, and Karlsson 2024; Chachula, Myrick, and Yonge 2015; Jarden et al. 2023; King and McInerney 2006; MacKusick and Minick 2010)	
Nurses expressed lack of flexibility in managing resources and work schedules (Bäckström, Pöder, and Karlsson 2024; Jarden et al. 2023; Lögde et al. 2018)	
Nurses were concerned about workplace safety due to increased exposure to pathogens (King and McInerney 2006)	
Nurses developed health problems related to work shifts (Lögde et al. 2018)	
Nurses reported dissatisfaction with the physical environment and insufficient organisational orientation towards staff well‐being (King and McInerney 2006)	
The lack of career growth opportunities and promotion chances led to frustration and low motivation	58%
Nurses experienced frustration and low motivation due to limited career opportunities and promotion chances (Bäckström, Pöder, and Karlsson 2024; Hung and Lam 2020; King and McInerney 2006; Kox et al. 2020; Pyhäjärvi and Söderberg 2024; Zhu et al. 2021)	
Nurses felt unfulfilled in their professional development needs and ambitions (Bäckström, Pöder, and Karlsson 2024; Hung and Lam 2020; Kox et al. 2020; Zhu et al. 2021)	
Nurses felt disappointed and tired of repeating the same work each day (Zhu et al. 2021)	
Nurses were disappointed by the disparity between the required effort and the opportunities for professional growth (Zhu, Rodgers, and Melia 2014)	
Disillusionment with nursing, encountering a gap between expectations and reality, with some lacking genuine passion, contributed to a bleak outlook on the future	58%
Nurses felt a discrepancy between their ideal perceptions of the nursing profession and the reality of their work experience, leading to disillusionment towards nursing (King and McInerney 2006; Kox et al. 2020; Pyhäjärvi and Söderberg 2024; Zhu, Rodgers, and Melia 2014)	
Nurses felt disappointed in the nursing profession (King and McInerney 2006; Pyhäjärvi and Söderberg 2024; Zhu, Rodgers, and Melia 2014)	
Nurses felt hopeless about the future (Chachula, Myrick, and Yonge 2015; MacKusick and Minick 2010; Pyhäjärvi and Söderberg 2024)	
Nurses felt lack of autonomy in their work (King and McInerney 2006; MacKusick and Minick 2010; Pyhäjärvi and Söderberg 2024)	
Nurses had the wrong motivation for entering the nursing profession and lacked passion for nursing care (King and McInerney 2006; Kox et al. 2020; Zhu, Rodgers, and Melia 2014)	
The hours of duty caused unhappiness among nurses (King and McInerney 2006; Zhu et al. 2021)	
Dissatisfaction due to salary not reflecting qualifications, responsibilities, and experience	42%
Nurses were dissatisfied with their salary and could not see any positive improvement in their salary development (Bäckström, Pöder, and Karlsson 2024; King and McInerney 2006; Kox et al. 2020; Lögde et al. 2018; Pyhäjärvi and Söderberg 2024)	
Nurses felt that their salary was not commensurate with their role, individual qualifications, responsibilities, and experience (Bäckström, Pöder, and Karlsson 2024; King and McInerney 2006; Pyhäjärvi and Söderberg 2024)	
Moral distress over ethical dilemmas and a perceived lack of interest in patient safety from managers	33%
Nurses experienced moral distress regarding ethical dilemmas in patient care (MacKusick and Minick 2010; Zhu, Rodgers, and Melia 2014)	
Nurses reported lack of interest in patient safety by managers (Bäckström, Pöder, and Karlsson 2024; Pyhäjärvi and Söderberg 2024)	

The most represented key meta‐finding, with an interstudy frequency effect size of 100%, was ‘Poor management practices, including lack of support, ineffective leadership, and unfair treatment within the healthcare organization’. Conversely, the less frequent key meta‐finding, with an interstudy frequency effect size of 33%, was ‘Moral distress over ethical dilemmas and a perceived lack of interest in patient safety from managers’. The study by Pyhäjärvi and Söderberg (2024) presented the highest intensity of ‘Statements of Findings’ (intrastudy intensity effect size of 100%), whereas the study by Zhu et al. (2021) reported the lowest (intrastudy intensity effect size of 33%), as presented in Table 5.

**TABLE 5:** Meta‐summary showing the intensity of every study used and the frequency of every key meta‐finding.

Key meta‐findings/studies	Bäckström, Pöder, and Karlsson (2024)	Çamveren, Arslan Yürümezoğlu, and Kocaman (2020)	Chachula, Myrick, and Yonge (2015)	Hung and Lam (2020)	Jarden et al. (2023)	King and McInerney (2006)	Kox et al. (2020)	Logde et al. (2018)	MacKusick and Minick (2010)	Pyhäjärvi and Söderberg (2024)	Zhu, Rodgers, and Melia (2014)	Zhu et al. (2021)	Interstudy frequency effect size^a^
Bullying, horizontal and vertical violence, gender‐based abuse, sexual harassment, patient and relative violence, and fear of repercussions for speaking out		X	X	X	X	X	X	X	X	X	X		10/12 (83%)
Disillusionment with nursing, encountering a gap between expectations and reality, with some lacking genuine passion, contributed to a bleak outlook on the future			X			X	X		X	X	X	X	7/12 (58%)
Dissatisfaction due to salary not reflecting qualifications, responsibilities, and experience continued	X					X	X	X		X			5/12 (42%)
Excessive workload, worsened by staff shortages, role ambiguity, COVID‐19, additional duties such as training, and unclear role boundaries	X	X	X	X	X	X	X	X	X	X	X		11/12 (92%)
Moral distress over ethical dilemmas and a perceived lack of interest in patient safety from managers	X								X	X	X		4/12 (33%)
Poor management practices, including lack of support, ineffective leadership, and unfair treatment within the healthcare organisation	X	X	X	X	X	X	X	X	X	X	X	X	12/12 (100%)
Teamwork hurdles, including lack of collaboration, support, understanding, negative relationships, senior resistance to change, and medical dominance	X		X	X	X	X	X	X	X	X	X		10/12 (83%)
The lack of career growth opportunities and promotion chances led to frustration and low motivation	X			X		X	X			X	X	X	7/12 (58%)
Health issues related to work shift and Difficulty in maintaining work‐life balance	X	X	X		X	X	X	X	X	X	X		10/12 (83%)
Intrastudy intensity effect size^b^	7/9 (78%)	4/9 (44%)	6/9 (67%)	5/9 (56%)	6/9 (67%)	8/9 (89%)	8/9 (89%)	6/9 (67%)	7/9 (78%)	9/9 (100%)	8/9 (89%)	3/9 (33%)	

^a^ Individual studies' contribution to key meta‐findings.

^b^ Representation of key meta‐findings in individual studies.

#### 3.2.1 Poor Management Practices, Including Lack of Support, Ineffective Leadership, and Unfair Treatment Within the Healthcare Organisation

Within this key meta‐finding, resigned nurses reported that they felt unsupported by managers in resolving workplace difficulties and endorsing their professional development (Bäckström, Pöder, and Karlsson 2024; Hung and Lam 2020; Jarden et al. 2023; King and McInerney 2006; MacKusick and Minick 2010; Pyhäjärvi and Söderberg 2024; Zhu, Rodgers, and Melia 2014; Zhu et al. 2021).

“… I was involved in a conflict incident [with a senior nurse] … I've never been in a situation like that in all my years of nursing, and I didn't really know what to do… [we had a discussion] in the manager's office…that was it, and then it would be over for another few months. And that happened again, and again, and again … HR [Human Resources] listened to her, and they listened to me. And they totally supported her, and totally did not support me … I feel like HR need to support both sides … there're two sides to every conflict…” (Jarden et al. 2023)

Poor management practices also affected the perceived competence of nurses, particularly junior ones who didn't receive proper onboarding (Çamveren, Arslan Yürümezoğlu, and Kocaman 2020; Kox et al. 2020) or nurses who were outsourced (Bäckström, Pöder, and Karlsson 2024). They described the feeling of not possessing adequate competence and experience regarding the specific condition of patients as especially stressful (Bäckström, Pöder, and Karlsson 2024). Regarding staff practice and towards managers, resigned nurses felt disappointed and dissatisfied (King and McInerney 2006; Kox et al. 2020; Pyhäjärvi and Söderberg 2024). Participants also felt undervalued and unappreciated in their experiences and competencies (Bäckström, Pöder, and Karlsson 2024; Hung and Lam 2020; Jarden et al. 2023; Kox et al. 2020). The inability of organisations to value professionals was also linked to the feeling that nurses had of being replaceable in the organisation (Lögde et al. 2018; Pyhäjärvi and Söderberg 2024; Zhu, Rodgers, and Melia 2014).

“I expected that my department or hospital would appreciate my efforts. However, this was merely wishful thinking. I was not as important as I thought I was. I did not feel that my department or the hospital treasured me as an effective staff member. I realised that what I had done was so meaningless. My intention to leave the profession was ignited at that moment.” (Hung and Lam 2020)

“Easiest for managers that all [nurses] are the same, just go in and do their job and nothing more. Do not ask any questions or show any personal interest. […].” (Bäckström, Pöder, and Karlsson 2024)

“Actually, they did not care to lose nurses; there are plenty of young nurses available to replace us.” (Zhu, Rodgers, and Melia 2014)

Resigned nurses reported an ineffective leadership style, which affected communication, a lack of listening to or ignoring their concerns, not being taken seriously, making decisions without employee involvement and not having the employees' best interests in mind (Bäckström, Pöder, and Karlsson 2024; Chachula, Myrick, and Yonge 2015; Hung and Lam 2020; Lögde et al. 2018).

In different studies, participants perceived unequal and unfair treatment regarding the allocation of role opportunities, duties, and shifts (Hung and Lam 2020; Jarden et al. 2023; King and McInerney 2006; Lögde et al. 2018) and reported subjective processes utilised in the performance appraisals (King and McInerney 2006).

#### 3.2.2 Excessive Workload, Worsened by Staff Shortages, Role Ambiguity, COVID‐19, Additional Duties Such as Training, and Unclear Role Boundaries Caused Stress and Frustration

An excessive workload was presented in 83% of the selected reports. Nurses reported that an excessive workload, mainly due to staff shortages, and continuous requests to work overtime led to their decision to leave the organisation (Bäckström, Pöder, and Karlsson 2024; Çamveren, Arslan Yürümezoğlu, and Kocaman 2020; Hung and Lam 2020; Kox et al. 2020; Lögde et al. 2018; MacKusick and Minick 2010; Pyhäjärvi and Söderberg 2024; Zhu, Rodgers, and Melia 2014).

“I thought that I couldn't work at this hospital at this pace until I retired. It is constantly exhausting, more and more workload, and has a lack of nurses ….” (Çamveren, Arslan Yürümezoğlu, and Kocaman 2020)

Besides staff shortages, the workload was significantly increased by the ambiguous scope of practice and unclear role boundaries (Hung and Lam 2020; Zhu, Rodgers, and Melia 2014). Additional duties, such as training new staff, negatively impacted the workload of junior nurses (Bäckström, Pöder, and Karlsson 2024; Hung and Lam 2020). Two studies reported how COVID‐19 negatively affected the workload of resigned nurses (Jarden et al. 2023; Pyhäjärvi and Söderberg 2024).

“My hospital frequently emphasized that we nurses had to treat our patients like our guests, and we should offer them warm hospitality and suggest that they make themselves at home. We were asked to handle the relatives' requests and complaints. Sometimes those requests were nonsense; perhaps they thought that they were the customers, and we were the waitresses. Time was spent and wasted in this manner. It is ridiculous.” (Hung and Lam 2020)

The intense workloads led nurses to experience significant stress, resulting in both psychological and physical symptoms, such as fatigue, sleep disturbances, headaches, muscle tension, lack of confidence and difficulty relaxing, which negatively impacted their health, well‐being, and ability to recover after work (Bäckström, Pöder, and Karlsson 2024; Hung and Lam 2020; Kox et al. 2020). Poor working conditions (Bäckström, Pöder, and Karlsson 2024; Lögde et al. 2018) and a stressful work environment led nurses to feel dissatisfied with their job (Chachula, Myrick, and Yonge 2015; Hung and Lam 2020; King and McInerney 2006).

The dissatisfaction with their work situation was exacerbated by concerns that stress and excessive workload could result in critical incidents and compromise patient safety (Bäckström, Pöder, and Karlsson 2024; Chachula, Myrick, and Yonge 2015; Kox et al. 2020; Pyhäjärvi and Söderberg 2024). Due to their intense workload, nurses were also frustrated at not being able to provide the quality of care they wished to deliver and felt like they had not done enough for patients (Bäckström, Pöder, and Karlsson 2024; Kox et al. 2020; Pyhäjärvi and Söderberg 2024; Zhu, Rodgers, and Melia 2014).

“You feel guilty also when you cannot care for the patients as well as you would want to.” (Pyhäjärvi and Söderberg 2024)

#### 3.2.3 Bullying, Horizontal and Vertical Violence, Gender‐Based Abuse, Sexual Harassment, Patient and Relative Violence, and Fear of Repercussions for Speaking out

Experiences of workplace violence were reported in 83% of the included studies. Resigned nurses had experienced bullying and horizontal violence at work (Çamveren, Arslan Yürümezoğlu, and Kocaman 2020; Chachula, Myrick, and Yonge 2015; King and McInerney 2006; Hung and Lam 2020; Lögde et al. 2018; Kox et al. 2020; MacKusick and Minick 2010; Pyhäjärvi and Söderberg 2024). These behaviours were predominantly carried out by senior colleagues towards junior colleagues (Çamveren, Arslan Yürümezoğlu, and Kocaman 2020; King and McInerney 2006; Lögde et al. 2018; Pyhäjärvi and Söderberg 2024) or by medical staff (Lögde et al. 2018; King and McInerney 2006). Two reports described stories of gender abuse and sexual harassment, with perceived acceptance of this behaviour by managers and colleagues (MacKusick and Minick 2010; Pyhäjärvi and Söderberg 2024).

“I wouldn't call it sexual harassment…It was just part and parcel with what you dealt with when we were…in the hospital. But it happened, and it was accepted, and essentially word got around that if you make rounds with doctor so and so [you should] make sure you are on the opposite side of the bed. You just sort of, you dealt with it.” (MacKusick and Minick 2010)

Resigned nurses had also experienced vertical violence at work (Çamveren, Arslan Yürümezoğlu, and Kocaman 2020; Zhu, Rodgers, and Melia 2014). Despite nurses' job satisfaction due to patient contact and interaction (King and McInerney 2006), they also experienced patient violence or violence by patients' relatives (King and McInerney 2006; Kox et al. 2020; Pyhäjärvi and Söderberg 2024). Patient violence contributed to a feeling of unsafety and, therefore, to an increase in the psychosocial workload (Kox et al. 2020).

#### 3.2.4 Teamwork Hurdles, Including Lack of Collaboration, Support, Understanding, Negative Relationships, Senior Resistance to Change, and Medical Dominance

Negative interpersonal relationships among colleagues were perceived as an important factor contributing to the decision to leave (interstudy frequency effect size of 83%) and causing workplace tension, dissatisfaction and a challenging work environment (Hung and Lam 2020; King and McInerney 2006; Kox et al. 2020; Lögde et al. 2018; Pyhäjärvi and Söderberg 2024). Nurses expressed a lack of team collaboration (Bäckström, Pöder, and Karlsson 2024; Zhu, Rodgers, and Melia 2014), particularly when they reported difficulties in dealing with medical dominance (Bäckström, Pöder, and Karlsson 2024; Chachula, Myrick, and Yonge 2015; King and McInerney 2006; Pyhäjärvi and Söderberg 2024; Zhu, Rodgers, and Melia 2014). They felt a lack of understanding, acceptance, and support from their colleagues (Bäckström, Pöder, and Karlsson 2024; Jarden et al. 2023; Kox et al. 2020; MacKusick and Minick 2010). Junior nurses also reported a reluctance to change by senior colleagues (Kox et al. 2020).

“How they [colleagues] behaved towards one another, it was a real revelation to me. Because it was, well, it was a women's world in fact. And that's what you feel, it's so nasty, it's so sly, so underhand, so….” (Kox et al. 2020)

“…And there was a very strong juxtaposition between the old and the new [employees] ‘…’ There was a fear that you couldn't say anything personal because it could then be used against you, and you could be targeted for negative attention if they didn't like your way of thinking or something.” (Pyhäjärvi and Söderberg 2024)

#### 3.2.5 Health Issues Related to Work Shifts and Difficulty in Maintaining Work‐Life Balance

Working hours and scheduling had played an important role in the decision to quit (Bäckström, Pöder, and Karlsson 2024). Nurses reported the negative effects that work shifts had on their private lives and the difficulty in maintaining work‐life balance (Bäckström, Pöder, and Karlsson 2024; Çamveren, Arslan Yürümezoğlu, and Kocaman 2020; King and McInerney 2006; Lögde et al. 2018; Kox et al. 2020; Pyhäjärvi and Söderberg 2024; Zhu, Rodgers, and Melia 2014). Concerning this struggle, they expressed a lack of flexibility in work schedules (Bäckström, Pöder, and Karlsson 2024; Jarden et al. 2023; Lögde et al. 2018) and the efforts to fulfil their social roles outside of the work context (Bäckström, Pöder, and Karlsson 2024; Çamveren, Arslan Yürümezoğlu, and Kocaman 2020; Pyhäjärvi and Söderberg 2024).

“When you become a mother, you really understand how precious time is; for example, I can't watch my child grow up, I'm constantly in the hospital; I can't spare any time for him.” (Çamveren, Arslan Yürümezoğlu, and Kocaman 2020)

“… The main problem is the hours. I have a young family, I have got three children one aged 6, one is nearly 4 and one is 2 and a half and …On the weekends when they wake up while I am creeping out in the morning to go to work… then they start crying because ‘why are you going to work – it's a weekend?’… I can't have a nursing career and a family with kids at the same time because shift work and family just do not mix….” (King and McInerney 2006)

“I felt … that you really had to sacrifice everything for the patient, your own life, just to get the operating schedule through. I do not think that it should be like that….” (Lögde et al. 2018)

They also reported insufficient organisational orientation towards staff well‐being (King and McInerney 2006). Health issues related to shifts were both physical (Lögde et al. 2018) and phycological (Bäckström, Pöder, and Karlsson 2024; Chachula, Myrick, and Yonge 2015; Jarden et al. 2023; King and McInerney 2006; MacKusick and Minick 2010). Five of the included studies reported resigned nurses' emotional and physical exhaustion (Bäckström, Pöder, and Karlsson 2024; Chachula, Myrick, and Yonge 2015; Jarden et al. 2023; King and McInerney 2006; MacKusick and Minick 2010).

“I worked days and nights, and it was killing me. The switching, the nights—it was just awful.” (Chachula, Myrick, and Yonge 2015)

#### 3.2.6 The Lack of Career Growth Opportunities and Promotion Chances Led to Frustration and Low Motivation

A significant factor in nurses tendering their resignations was the perception of limited career opportunities available in nursing (Pyhäjärvi and Söderberg 2024) or within specific organisations (Zhu et al. 2021). Low promotion chances led them to experience frustration and low motivation (Bäckström, Pöder, and Karlsson 2024; Hung and Lam 2020; King and McInerney 2006; Kox et al. 2020; Pyhäjärvi and Söderberg 2024; Zhu et al. 2021). Nurses felt disappointed and tired of repeating the same work each day (Zhu et al. 2021) and felt unfulfilled in their professional development needs and ambitions (Bäckström, Pöder, and Karlsson 2024; Hung and Lam 2020; Kox et al. 2020; Zhu et al. 2021). In addition, nurses were disappointed by the disparity between the required effort and the opportunities for professional growth (Zhu, Rodgers, and Melia 2014).

“I have been chasing my own tail nearly a year. I have seen no sign of promotion … I find no value in working. My gain is nothing compared with my effort (repeated). Now I can see no flicker of hope.” (Zhu et al. 2021)

#### 3.2.7 Disillusionment With Nursing and Encountering a Gap Between Expectations and Reality, With Some Nurses' Lacking Genuine Passion, Contributed to a Bleak Outlook on the Future

The lack of core motivation was reported in 50% of the included studies. While some nurses had entered the profession for the wrong reasons and were not truly passionate about nursing (King and McInerney 2006; Kox et al. 2020; Zhu, Rodgers, and Melia 2014), other nurses soon felt a discrepancy between their ideal thoughts on nursing and the reality of their work experience. This perceived discrepancy led them to become disillusioned with nursing (King and McInerney 2006; Kox et al. 2020; Pyhäjärvi and Söderberg 2024; Zhu, Rodgers, and Melia 2014), disappointed (King and McInerney 2006; Pyhäjärvi and Söderberg 2024; Zhu, Rodgers, and Melia 2014), and pessimistic about the future (Chachula, Myrick, and Yonge 2015; MacKusick and Minick 2010; Pyhäjärvi and Söderberg 2024). Resigned nurses complained about the lack of autonomy in their work (King and McInerney 2006; MacKusick and Minick 2010; Pyhäjärvi and Söderberg 2024).

“In practice, you have to perform every role; you're a phone operator and you have to make the beds and you have to help patients go to the toilet, and yes. Just a lot of minor tasks. That was ultimately not my ambition. […] At one point I thought, it feels a bit like a dead end, and then what? Being a nurse, OK, and that's it. Yes, I think that is in fact the difference between what you are trained for and what you really do in practice.” (Kox et al. 2020)

#### 3.2.8 Dissatisfaction Due to Salary Not Reflecting Qualifications, Responsibilities, and Experience

Being dissatisfied with their salary was reported in 42% of the included studies. Specifically, resigned nurses did not see any positive improvement in their salary development (Bäckström, Pöder, and Karlsson 2024; King and McInerney 2006; Kox et al. 2020; Lögde et al. 2018; Pyhäjärvi and Söderberg 2024) and felt that their salary was not commensurate with their role, individual qualifications, responsibilities, and experience (Bäckström, Pöder, and Karlsson 2024; King and McInerney 2006; Pyhäjärvi and Söderberg 2024).

“Well, I was really annoyed by the fact that I had learned a second demanding specialty … And I then tried to get into this higher pay level. … I remember when I heard the decision that I couldn't get even the 70€ increase … it was like the last drop.” (Pyhäjärvi and Söderberg 2024)

#### 3.2.9 Moral Distress Over Ethical Dilemmas and a Perceived Lack of Interest in Patient Safety From Managers

Moral distress arises from 33% of studies. It was related to ethical dilemmas in patient care due to overly aggressive treatment, a lack of collaboration between physicians and staff, and a lack of respect for patient and family wishes (MacKusick and Minick 2010; Zhu, Rodgers, and Melia 2014). Resigned nurses also reported an ethically straight lack of interest in patient safety by managers (Bäckström, Pöder, and Karlsson 2024; Pyhäjärvi and Söderberg 2024).

“I remember near the end…I was crying, crying almost every day… I mean, what kind of job do you have where you cry every day? That is when I knew, when I had to look for another job.” (MacKusick and Minick 2010)

## 4 Discussion

This synthesis of qualitative studies and meta‐summary aimed to provide an overview of the motivations that have led nurses to resign from healthcare organisations or leave the profession. It not only examined the widely discussed phenomenon of the intention to leave but also delved into the lived experiences of professionals. Additionally, it investigated those factors that, when associated with the intention to quit, were most involved in the actual departure from the organisation or the profession.

The meta‐summary included studies from various countries with significant differences in healthcare system organisation, covering a broad time span from 2006 (King and McInerney 2006) to 2024 (Bäckström, Pöder, and Karlsson 2024; Pyhäjärvi and Söderberg 2024). For instance, in Turkey, nurses historically had permanent employment in public hospitals, which provided job security until retirement. However, the 2003 Health Transformation Program led to many being employed under contracts, which reduced their employee benefits and increased job dissatisfaction (Çamveren, Arslan Yürümezoğlu, and Kocaman 2020). Furthermore, in Hong Kong, the ongoing culture of the healthcare sector reveals chronic issues with staff turnover and shortages. The Hospital Authority of Hong Kong, which manages 43 public hospitals, has expressed deep concern over the overwhelming workloads faced by frontline nurses, as nurse‐to‐patient ratios during night shifts significantly exceed international standards (Hung and Lam 2020). Despite the differences in time and location, these studies showed a recurrence of findings. This suggests that the reasons why nurses leave organisations are less influenced by the geographical context and are more related to the intrinsic characteristics of the nursing profession and the organisational dynamics of healthcare organisations. Although it can be assumed that significant changes have occurred over nearly 20 years, both in terms of professional development and organisation, the main findings emerge with surprising continuity even after many years.

Three of the included studies referred solely to leaving the organisation (Bäckström, Pöder, and Karlsson 2024; Çamveren, Arslan Yürümezoğlu, and Kocaman 2020; Lögde et al. 2018). Four studies, on the other hand, focused on leaving the profession after resigning from hospital (Chachula, Myrick, and Yonge 2015; Hung and Lam 2020; Kox et al. 2020; Pyhäjärvi and Söderberg 2024). Two studies included participants who had both left their organisations and the profession (Jarden et al. 2023; King and McInerney 2006). Zhu, Rodgers, and Melia (2014) and MacKusick and Minick (2010) included nurses who left clinical nursing, whereas Zhu et al. (2021) included nurses who resigned without specifying if they also abandoned the profession. Although leaving the organisation and leaving the profession can be considered two separate phenomena, in this synthesis, they are understood as two final steps of the same process and, for this reason, investigated together. The phenomenon of turnover develops on a continuum and progressively evolves from milder to more severe forms; leaving the organisation is seen as the most severe manifestation of turnover, which can lead to leaving the profession (Krausz et al. 1995).

Three of the included reports focused on turnover among novice nurses, with millennials as participants (Çamveren, Arslan Yürümezoğlu, and Kocaman 2020; Chachula, Myrick, and Yonge 2015; Kox et al. 2020). Within these reports, recurring findings emerge, such as difficulty maintaining work‐life balance, managing excessive workloads, experiencing bullying, and perceiving a lack of support from managers and colleagues. These findings resonate with broader literature on the matter. Climek, Henry, and Jeong (2022) observed that millennials prioritise work‐life balance and are more likely to leave positions that do not meet this need. Additionally, they tend to seek jobs that offer personal satisfaction and autonomy, rather than solely focusing on external rewards. Furthermore, Climek, Henry, and Jeong (2022) highlighted that millennials are more likely to remain in their jobs when they have opportunities for social interaction and meaningful relationships with colleagues (Climek, Henry, and Jeong 2022).

Our findings are coherent with the results found in previous reviews on the intention to leave phenomenon (Bahlman‐van Ooijen et al. 2023; Coomber and Barriball 2007; Hayes et al. 2012), which was identified as a predictor of turnover (Hayes et al. 2006). Nurse turnover is influenced by multiple determinants. The analysis of qualitative studies revealed how these are strongly connected. The findings indicated that the final decision to leave was not due to a single motivation but rather to a combination of factors that cumulatively led to the decision. Although it was not possible to identify which factor was the decisive trigger, the analysis of the frequency effects of the findings allowed for determining their recurrence in the studies.

All selected studies presented poor management as a finding. Management practices influenced the decision to resign in many ways: failing to support nurses in resolving work difficulties (Bäckström, Pöder, and Karlsson 2024; Hung and Lam 2020; King and McInerney 2006); neglecting their professional development (Zhu et al. 2021) and affecting the way nurses perceived themselves, as less competent (Bäckström, Pöder, and Karlsson 2024; Çamveren, Arslan Yürümezoğlu, and Kocaman 2020; Kox et al. 2020; MacKusick and Minick 2010; Pyhäjärvi and Söderberg 2024), undervalued (Bäckström, Pöder, and Karlsson 2024; Hung and Lam 2020; Jarden et al. 2023; Kox et al. 2020), replaceable (Lögde et al. 2018; Pyhäjärvi and Söderberg 2024; Zhu, Rodgers, and Melia 2014), and unheard (Hung and Lam 2020; Jarden et al. 2023). These findings indicated that nursing managers may have some responsibility for the flight of nurses. At a higher level, the organisation, administrators and even the policies and regulations governing the nursing profession can be perceived as insensitive and unsupportive of nurses' mission, leading them to feel discouraged and quit the profession (Tuckett et al. 2015). Although high‐level managers often operate within the constraints of limited resources and face complex challenges given by the rapid evolution of healthcare systems (Figueroa et al. 2019), it is essential that they still commit to adopt policies oriented towards staff well‐being, valuing and recognising the health professionals' profiles and roles.

Several managerial factors affected the decision to leave organisations or the profession. The inflexibility of work shifts made it hard to maintain work‐life balance and fulfil nurses' social roles outside of work (Bäckström, Pöder, and Karlsson 2024; Çamveren, Arslan Yürümezoğlu, and Kocaman 2020; King and McInerney 2006; Lögde et al. 2018; Kox et al. 2020; Pyhäjärvi and Söderberg 2024; Zhu, Rodgers, and Melia 2014). Work‐life balance was also affected by excessive workload and by requests to work overtime. This led nurses to experience emotional and physical exhaustion (Bäckström, Pöder, and Karlsson 2024; Chachula, Myrick, and Yonge 2015; Jarden et al. 2023; King and McInerney 2006; MacKusick and Minick 2010). Several nurses claimed they left the clinical practice to improve their well‐being and quality of family life (Zhu, Rodgers, and Melia 2014).

Resigned nurses reported that an excessive workload, mainly due to staff shortages, supported their decision to leave (Bäckström, Pöder, and Karlsson 2024; Çamveren, Arslan Yürümezoğlu, and Kocaman 2020; Hung and Lam 2020; Kox et al. 2020; Lögde et al. 2018; MacKusick and Minick 2010; Pyhäjärvi and Söderberg 2024; Zhu, Rodgers, and Melia 2014). Two of the included studies cited COVID‐19 as a source of additional workload (Jarden et al. 2023; Pyhäjärvi and Söderberg 2024). Indeed, COVID‐19 was considered a cause of the intensification of preexisting problems rather than being the main cause of resignation (Pyhäjärvi and Söderberg 2024). While it has been shown that excessive workload is a factor in nursing turnover, a high‐demand work situation does not necessarily lead to turnover unless other conditions also occur (Hayes et al. 2012). Turnover is more likely to happen when work demands are associated with a lack of control over work, a lack of support from the team, and other resources, and when work becomes so demanding, both physically and mentally, that nurses perceive they are too busy to provide good standards of care (Hayes et al. 2012). Findings from the meta‐summary show that resigned nurses felt frustrated and guilty about not to being able to provide their patients with the quality care they wished to deliver (Bäckström, Pöder, and Karlsson 2024; Kox et al. 2020; Pyhäjärvi and Söderberg 2024; Zhu, Rodgers, and Melia 2014).

Dissatisfaction emerged as a crucial and cross‐cutting factor in the meta‐summary's findings, mainly related to heavy workload (Kox et al. 2020), staffing practice (King and McInerney 2006), relationships with colleagues (Hung and Lam 2020; King and McInerney 2006) and salary (Bäckström, Pöder, and Karlsson 2024; King and McInerney 2006; Kox et al. 2020; Lögde et al. 2018; Pyhäjärvi and Söderberg 2024). Job satisfaction is considered in the literature to be one of the main determinants of voluntary nurse turnover (De Simone, Planta, and Cicotto 2018). When nurses' job satisfaction decreases, their intention to quit increases (Hayes et al. 2012). This means that creating favourable working conditions aimed at stimulating a sense of satisfaction through the recognition of one's personal values related to work could reduce the likelihood of leaving the organisation (De Simone, Planta, and Cicotto 2018).

The organisational environment is influenced by interpersonal relationships among colleagues. Nurses who have left the profession have often experienced a rigid hierarchy among nurses, frequently leading to horizontal violent behaviours such as bullying (Çamveren, Arslan Yürümezoğlu, and Kocaman 2020; Chachula, Myrick, and Yonge 2015; King and McInerney 2006; Hung and Lam 2020; Lögde et al. 2018; Kox et al. 2020; MacKusick and Minick 2010; Pyhäjärvi and Söderberg 2024). Bullying within the nursing culture is currently a widespread issue, with as many as 75%–85% of nurses potentially experiencing verbal or non‐verbal bullying from managers or colleagues during their careers (Bahlman‐van Ooijen et al. 2023). Violence in the workplace not only creates a hostile atmosphere but also compromises nurses' psychological well‐being. What is particularly concerning is that resigned nurses perceived a lack of support from managers. In many cases, they even had the impression that such behaviours were tolerated or even accepted by the leadership (Pyhäjärvi and Söderberg 2024) and by their colleagues (MacKusick and Minick 2010).

### 4.1 Strength and Limitations

Currently, this research constitutes the first meta‐summary of qualitative studies specifically focused on the motivation behind nurses' resignations and intentions to leave the profession. To enhance validity, various strategies were used. A web‐based software was utilised to systematically screen titles and abstracts, a process independently carried out by two researchers. Any discrepancies in judgements regarding the inclusion or exclusion of full‐text articles were resolved through a consensus process. Subsequently, report characteristics were extracted using a standardised data extraction sheet.

This synthesis has several limitations. One notable limitation of our study is the decision not to define a specific time frame for the included research studies. While this approach was intended to capture a broad range of relevant literature, it may include studies that are less applicable to the current nursing situation. Although our results indicate that the motivations for leaving have remained similar over time, the absence of a defined time frame introduces a potential variability in the relevance of some findings. Then, the number of studies included in the synthesis is limited. This may be attributed to the difficulty in obtaining samples from resigned nurses and to the frequent use in the literature of intention to leave as a predictive indicator for turnover. Moreover, some studies did not distinguish between those who actually left the profession and those who simply left the hospital. Future research should investigate these two populations separately. In addition, since studies from various countries were included in the meta‐summary and some of them are not by native English‐speakers, there may be some differences in the interpretation of the reported findings. Moreover, the search included only studies published in English. Thus, data not published in English are likely to have been overlooked. Finally, the meta‐summary combined studies used different methodological approaches. This can be seen as a limitation since combining different methods is a controversial issue in the literature (Mohammed and Moles 2016). In fact, these differing study designs lead to variations in how data are gathered, analysed, and understood, making it challenging to integrate findings from studies using distinct methodological approaches. However, in our synthesis, most of the studies are descriptive in design.

### 4.2 Implication for Research and Practice

Despite the limited number of qualitative studies focusing on the phenomenon and the lack of representativeness in various contexts, the findings of this research highlighted the underlying motivations behind nurses' final decision to leave. Those reasons seemed consistent with the available literature on factors influencing the intention to leave at the time of the current study. The findings appeared to have shown minimal variation over time.

Specific implications for both practice and research emerged. Regarding implications for research, a more in‐depth investigation into the generational aspects related to turnover would be expected, with particular attention directed towards the recent entry of Generation Z into the workforce. At the same time, future research should address the motivations that drive nurses to remain within organisations for extended periods. Lastly, a clear distinction between the intention to leave an organisation and the intention to leave the nursing profession should be made in primary studies. Separating these concepts will enable more targeted interventions and policies to address both organisational issues and broader systemic challenges in nursing.

Regarding practical implications, the shortage of nursing staff is a longstanding global issue that demands effective retention strategies at both organisational and political levels (NSI Nursing Solutions Inc 2024). Nurse managers must identify and monitor the specific factors influencing nurse turnover within their organisations. Enhancing nurse managers' leadership skills through targeted training programs is therefore crucial. Based on the findings of this synthesis, healthcare organisations should implement tailored measures to prevent nurse turnover, adapting these strategies to the unique characteristics of each organisation. For instance, in high‐stress environments, introducing stress management programs and flexible scheduling can help reduce burnout. In areas with high turnover rates, improving onboarding processes and offering robust career development opportunities can enhance job satisfaction and retention. Additionally, providing organisational support through mentoring and preceptorship programs, and promoting career pathways that ensure equitable professional development opportunities are essential. Healthcare organisations and governments should also focus on managing nurses' workloads effectively to minimise dissatisfaction and burnout, while increasing work flexibility to promote a better work‐life balance. Lastly, fostering a respectful and supportive work environment with zero‐tolerance policies for violence and bullying, and offering support networks for nurses experiencing distress, are crucial strategies for improving nurses' work experience.

## 5 Conclusion

This meta‐summary provides an overview of the evidence from qualitative studies exploring the experiences of nurses who have left healthcare organisations or abandoned the profession. ‘Poor management practices, including lack of support, ineffective leadership and unfair treatment within healthcare organizations’, was the most recorded finding. Other key meta‐findings include: excessive workloads; teamwork hurdles; health issues related to work shifts and difficulty in maintaining work‐life balance; bullying and workplace violence; lack of career growth opportunities and promotion chances, disillusionment with nursing, dissatisfaction with salaries, moral distress over ethical dilemmas.

The shortage of nurses is an increasingly prevalent and multifactorial problem that requires diversified interventions to address it. Healthcare organisations cannot afford to lose nurses and must develop strategies to retain these professionals within the organisations and, above all, within the profession. The findings of this synthesis can help to support the development of targeted strategies and the implementation of effective policies aimed at reducing nursing turnover.

## Author Contributions

Made substantial contributions to conception and design, or acquisition of data, or analysis and interpretation of data: L.L., I.B., M.D. Involved in drafting the manuscript or revising it critically for important intellectual content: L.L., M.D. Given final approval of the version to be published. Each author should have participated sufficiently in the work to take public responsibility for appropriate portions of the content: L.L., I.B., M.D. Agreed to be accountable for all aspects of the work in ensuring that questions related to the accuracy or integrity of any part of the work are appropriately investigated and resolved: L.L., I.B., M.D.

## Conflicts of Interest

The authors declare no conflicts of interest.

### Peer Review

The peer review history for this article is available at https://www.webofscience.com/api/gateway/wos/peer‐review/10.1111/jan.16546.

## Data Availability

Data sharing not applicable to this article as no datasets were generated or analysed during the current study.

## References

[jan16546-bib-0001] Aiken, L. H. , D. M. Sloane , L. Bruyneel , et al. 2014. “Nurse Staffing and Education and Hospital Mortality in Nine European Countries: A Retrospective Observational Study.” Lancet 383, no. 9931: 1824–1830. 10.1016/S0140-6736(13)62631-8. 24581683 PMC4035380

[jan16546-bib-0002] Alsubhi, H. , P. Meskell , D. O. Shea , and O. Doody . 2020. “Missed Nursing Care and Nurses' Intention to Leave: An Integrative Review.” Journal of Nursing Management 28, no. 8: 1830–1840. 10.1111/jonm.13069. 32526799

[jan16546-bib-0004] Bäckström, J. , U. Pöder , and A. C. Karlsson . 2024. “I Was Merely a Brick in the Game: A Qualitative Study on Registered Nurses' Reasons for Quitting Their Jobs in Hospitals.” Journal of Nursing Management 2024: 6662802. 10.1155/2024/6662802 PMC11919021 40224742

[jan16546-bib-0005] Bahlman‐van Ooijen, W. , S. Malfait , G. Huisman‐de Waal , and T. B. Hafsteinsdóttir . 2023. “Nurses' Motivations to Leave the Nursing Profession: A Qualitative Meta‐Aggregation.” Journal of Advanced Nursing 79, no. 12: 4455–4471. 10.1111/jan.15696. 37209086

[jan16546-bib-0006] Buchan, J. , H. Catton , and F. Shaffer . 2022. “Sustain and Retain in 2022 and Beyond.” The Global Nursing Workforce and the COVID‐19 Pandemic. Accessed May 18, 2024. https://www.icn.ch/resources/publications‐and‐reports/sustain‐and‐retain‐2022‐and‐beyond.

[jan16546-bib-0007] Çamveren, H. , H. Arslan Yürümezoğlu , and G. Kocaman . 2020. “Why Do Young Nurses Leave Their Organization? A Qualitative Descriptive Study.” International Nursing Review 67, no. 4: 519–528. 10.1111/inr.12633. 33006181

[jan16546-bib-0008] Chachula, K. M. , F. Myrick , and O. Yonge . 2015. “Letting Go: How Newly Graduated Registered Nurses in Western Canada Decide to Exit the Nursing Profession.” Nurse Education Today 35, no. 7: 912–918. 10.1016/j.nedt.2015.02.024. 25862074

[jan16546-bib-0009] Climek, M. , R. Henry , and S. Jeong . 2022. “Integrative Literature Review on Employee Turnover Antecedents Across Different Generations: Commonalities and Uniqueness.” European Journal of Training and Development 48, no. 1/2: 112–132. 10.1108/EJTD-05-2021-0058.

[jan16546-bib-0010] Cooke, A. , D. Smith , and A. Booth . 2012. “Beyond PICO: The SPIDER Tool for Qualitative Evidence Synthesis.” Qualitative Health Research 22, no. 10: 1435–1443. 10.1177/1049732312452938. 22829486

[jan16546-bib-0011] Coomber, B. , and K. L. Barriball . 2007. “Impact of Job Satisfaction Components on Intent to Leave and Turnover for Hospital‐Based Nurses: A Review of the Research Literature.” International Journal of Nursing Studies 44, no. 2: 297–314. 10.1016/j.ijnurstu.2006.02.004. 16631760

[jan16546-bib-0012] CREA . 2022. “Centro per la ricerca economica applicata in sanità.” Sanità—Rapporto Sanità 2022—Edizione XVIII. CREA Sanità. Accessed November 23, 2023. https://www.creasanita.it/attivitascientifiche/rapporto‐sanita‐2022‐edizione‐xviii/.

[jan16546-bib-0013] Critical Appraisal Skills Programme . 2023. “CASP Qualitative Studies Checklist.” Accessed May 15, 2024. https://casp‐uk.net/casp‐tools‐checklists/.

[jan16546-bib-0014] Dall'Ora, C. , C. Saville , B. Rubbo , L. Turner , J. Jones , and P. Griffiths . 2022. “Nurse Staffing Levels and Patient Outcomes: A Systematic Review of Longitudinal Studies.” International Journal of Nursing Studies 134: 104311. 10.1016/j.ijnurstu.2022.104311. 35780608

[jan16546-bib-0015] Danielis, M. , F. Movio , G. Milanese , and E. Mattiussi . 2024. “Patients' Reports on Their Delusional Memories From the Intensive Care Unit: A Systematic Review of Qualitative Studies.” Intensive & Critical Care Nursing 81: 103617. 10.1016/j.iccn.2023.103617. 38176133

[jan16546-bib-0016] De Simone, S. , A. Planta , and G. Cicotto . 2018. “The Role of Job Satisfaction, Work Engagement, Self‐Efficacy and Agentic Capacities on Nurses' Turnover Intention and Patient Satisfaction.” Applied Nursing Research 39: 130–140. 10.1016/j.apnr.2017.11.004. 29422148

[jan16546-bib-0019] Figueroa, C. A. , R. Harrison , A. Chauhan , and L. Meyer . 2019. “Priorities and Challenges for Health Leadership and Workforce Management Globally: A Rapid Review.” BMC Health Services Research 19, no. 1: 239. 10.1186/s12913-019-4080-7. 31014349 PMC6480808

[jan16546-bib-0020] Flinkman, M. , U. Isopahkala‐Bouret , and S. Salanterä . 2013. “Young Registered Nurses' Intention to Leave the Profession and Professional Turnover in Early Career: A Qualitative Case Study.” International Scholarly Research Notices 2013: e916061. 10.1155/2013/916061. PMC3762080 24027640

[jan16546-bib-0021] Flinkman, M. , H. Leino‐Kilpi , and S. Salanterä . 2010. “Nurses' Intention to Leave the Profession: Integrative Review.” Journal of Advanced Nursing 66, no. 7: 1422–1434. 10.1111/j.1365-2648.2010.05322.x. 20497270

[jan16546-bib-0023] Hayes, L. J. , L. O'Brien‐Pallas , C. Duffield , et al. 2012. “Nurse Turnover: A Literature Review—An Update.” International Journal of Nursing Studies 49, no. 7: 887–905. 10.1016/j.ijnurstu.2011.10.001. 22019402

[jan16546-bib-0024] Hayes, L. J. , L. O'Brien‐Pallas , C. Duffield , et al. 2006. “Nurse Turnover: A Literature Review.” International Journal of Nursing Studies 43, no. 2: 237–263. 10.1016/j.ijnurstu.2005.02.007. 15878771

[jan16546-bib-0025] Herber, O. R. , B. Bücker , M.‐I. Metzendorf , and J. Barroso . 2017. “A Qualitative Meta‐Summary Using Sandelowski and Barroso's Method for Integrating Qualitative Research to Explore Barriers and Facilitators to Self‐Care in Heart Failure Patients.” European Journal of Cardiovascular Nursing 16, no. 8: 662–677. 10.1177/1474515117711007. 28509565

[jan16546-bib-0026] Hung, M. S. Y. , and S. K. K. Lam . 2020. “Antecedents and Contextual Factors Affecting Occupational Turnover Among Registered Nurses in Public Hospitals in Hong Kong: A Qualitative Descriptive Study.” International Journal of Environmental Research and Public Health 17, no. 11: 3834. 10.3390/ijerph17113834. PMC7312687 32481664

[jan16546-bib-0027] Jarden, R. J. , S. Scott , N. Rickard , et al. 2023. “Factors Contributing to Nurse Resignation During COVID‐19: A Qualitative Descriptive Study.” Journal of Advanced Nursing 79, no. 7: 2484–2501. 10.1111/jan.15596. 36805610

[jan16546-bib-0029] King, L. A. , and P. A. McInerney . 2006. “Hospital Workplace Experiences of Registered Nurses That Have Contributed to Their Resignation in the Durban Metropolitan Area.” Curationis 29, no. 4: 70–81. 17310747

[jan16546-bib-0030] Kox, J. H. A. M. , J. H. Groenewoud , E. J. M. Bakker , et al. 2020. “Reasons Why Dutch Novice Nurses Leave Nursing: A Qualitative Approach.” Nurse Education in Practice 47: 102848. 10.1016/j.nepr.2020.102848. 32781415

[jan16546-bib-0031] Krausz, M. , M. Koslowsky , N. Shalom , and N. Elyakim . 1995. “Predictors of Intentions to Leave the Ward, the Hospital, and the Nursing Profession: A Longitudinal Study.” Journal of Organizational Behaviour 16: 277–288. 10.1002/job.4030160308.

[jan16546-bib-0033] Lögde, A. , G. Rudolfsson , R. R. Broberg , A. Rask‐Andersen , R. Wålinder , and E. Arakelian . 2018. “I Am Quitting My Job. Specialist Nurses in Perioperative Context and Their Experiences of the Process and Reasons to Quit Their Job.” International Journal for Quality in Health Care 30, no. 4: 303–320. Scopus. 10.1093/intqhc/mzy023. 29518200

[jan16546-bib-0034] MacKusick, C. I. , and P. Minick . 2010. “Why Are Nurses Leaving? Findings From an Initial Qualitative Study on Nursing Attrition.” Medsurg Nursing 19, no. 6: 335–340. 21337990

[jan16546-bib-0035] Methley, A. M. , S. Campbell , C. Chew‐Graham , R. McNally , and S. Cheraghi‐Sohi . 2014. “PICO, PICOS and SPIDER: A Comparison Study of Specificity and Sensitivity in Three Search Tools for Qualitative Systematic Reviews.” BMC Health Services Research 14: 579. 10.1186/s12913-014-0579-0. 25413154 PMC4310146

[jan16546-bib-0036] Mohammadi, P. , S. F. Gheiasi , R. Bayat , G. Bulfone , and K. Amini . 2023. “Nurses' Intention to Leave the Profession and Its Related Factors: A Cross‐Sectional Study.” Evidence Based Care 12, no. 4: 62–71. 10.22038/ebcj.2022.67012.2756.

[jan16546-bib-0037] Mohammed, M. , and R. Moles . 2016. “Meta‐Synthesis of Qualitative Research: The Challenges and Opportunities.” International Journal of Clinical Pharmacy 38: 695–704. 10.1007/s11096-016-0289-2. 27052213

[jan16546-bib-0038] NSI Nursing Solutions Inc . 2024. 2024 NSI National Health Care Retention & RN Staffing Report. NSI Nursing Solutions, Inc. https://www.nsinursingsolutions.com/Documents/Library/NSI_National_Health_Care_Retention_Report.pdf.

[jan16546-bib-0039] OECD & European Union . 2022. Health at a Glance: Europe 2022: State of Health in the EU Cycle. OECD. 10.1787/507433b0-en.

[jan16546-bib-0040] Pyhäjärvi, D. , and C. B. Söderberg . 2024. “The Straw That Broke the Nurse's Back—Using Psychological Contract Breach to Understand Why Nurses Leave.” Journal of Advanced Nursing 80, no. 12: 4989–5002. 38444207 10.1111/jan.16143

[jan16546-bib-0041] Raso, R. , J. J. Fitzpatrick , and K. Masick . 2021. “Nurses' Intent to Leave Their Position and the Profession During the COVID‐19 Pandemic.” JONA: The Journal of Nursing Administration 51, no. 10: 488–494. 10.1097/NNA.0000000000001052. 34519700

[jan16546-bib-0042] Sandelowski, M. , and J. Barroso . 2003. “Creating Metasummaries of Qualitative Findings.” Nursing Research 52, no. 4: 226–233. 10.1097/00006199-200307000-00004. 12867779

[jan16546-bib-0043] Sandelowski, M. , and J. Barroso . 2007. Handbook for Synthesizing Qualitative Research. New York: Springer Publishing Company.

[jan16546-bib-0044] Sasso, L. , A. Bagnasco , G. Catania , et al. 2019. “Push and Pull Factors of Nurses' Intention to Leave.” Journal of Nursing Management 27, no. 5: 946–954. 10.1111/jonm.12745. 30614593

[jan16546-bib-0045] Shin, S. , J.‐H. Park , and S.‐H. Bae . 2018. “Nurse Staffing and Nurse Outcomes: A Systematic Review and Meta‐Analysis.” Nursing Outlook 66, no. 3: 273–282. 10.1016/j.outlook.2017.12.002. 29685321

[jan16546-bib-0046] Tadesse, B. , A. Dechasa , M. Ayana , and M. R. Tura . 2023. “Intention to Leave Nursing Profession and Its Associated Factors Among Nurses: A Facility Based Cross‐Sectional Study.” INQUIRY: The Journal of Health Care Organization, Provision, and Financing 60: 00469580231200602. 10.1177/00469580231200602. PMC10521272 37746703

[jan16546-bib-0047] Tong, A. , K. Flemming , E. McInnes , S. Oliver , and J. Craig . 2012. “Enhancing Transparency in Reporting the Synthesis of Qualitative Research: ENTREQ.” BMC Medical Research Methodology 12: 181. 10.1186/1471-2288-12-181. 23185978 PMC3552766

[jan16546-bib-0048] Tuckett, A. , P. Winters‐Chang , F. Bogossian , and M. Wood . 2015. “«Why Nurses Are Leaving the Profession … Lack of Support From Managers»: What Nurses From an e‐Cohort Study Said.” International Journal of Nursing Practice 21, no. 4: 359–366. 10.1111/ijn.12245. 24571860

[jan16546-bib-0049] Tummers, L. G. , S. M. Groeneveld , and M. Lankhaar . 2013. “Why Do Nurses Intend to Leave Their Organization? A Large‐Scale Analysis in Long‐Term Care.” Journal of Advanced Nursing 69, no. 12: 2826–2838. 10.1111/jan.12249. 24016210

[jan16546-bib-0050] Ulupınar, F. , and Y. Erden . 2024. “Intention to Leave Among Nurses During the COVID‐19 Outbreak: A Rapid Systematic Review and Meta‐Analysis.” Journal of Clinical Nursing 33, no. 1: 393–403. 10.1111/jocn.16588. 36435976

[jan16546-bib-0051] Wang, L. , H. Lu , X. Dong , et al. 2020. “The Effect of Nurse Staffing on Patient‐Safety Outcomes: A Cross‐Sectional Survey.” Journal of Nursing Management 28, no. 7: 1758–1766. 10.1111/jonm.13138. 32853457

[jan16546-bib-0052] Winter, V. , J. Schreyögg , and A. Thiel . 2020. “Hospital Staff Shortages: Environmental and Organizational Determinants and Implications for Patient Satisfaction.” Health Policy 124, no. 4: 380–388. 10.1016/j.healthpol.2020.01.001. 31973906

[jan16546-bib-0053] World Health Organization . 2020. State of the World's Nursing 2020: Investing in Education, Jobs and Leadership. World Health Organization. https://iris.who.int/handle/10665/331677.

[jan16546-bib-0054] Zhu, H. , C. Xu , H. Jiang , and M. Li . 2021. “A Qualitative Study on the Experiences and Attributions for Resigned Nurses With Career Plateau.” International Journal of Nursing Studies 8, no. 3: 325–331. 10.1016/j.ijnss.2021.05.006. PMC8283717 34307782

[jan16546-bib-0055] Zhu, J. , S. Rodgers , and K. M. Melia . 2014. “A Qualitative Exploration of Nurses Leaving Nursing Practice in China.” Nursing Open 2, no. 1: 3–13. 10.1002/nop2.11. 27708796 PMC5047306

